# Enhancing activities of daily living of chronic stroke patients in primary health care by modified constraint-induced movement therapy (HOMECIMT): study protocol for a cluster randomized controlled trial

**DOI:** 10.1186/1745-6215-14-334

**Published:** 2013-10-14

**Authors:** Anne Barzel, Gesche Ketels, Britta Tetzlaff, Heike Krüger, Kerstin Haevernick, Anne Daubmann, Karl Wegscheider, Martin Scherer

**Affiliations:** 1 Department of Primary Medical Care, University Medical Center Hamburg-Eppendorf, Martinistr. 52, 20246 Hamburg, Germany; 2 Physiotherapy, University Medical Center Hamburg-Eppendorf, Martinistraße 52, 20246 Hamburg, Germany; 3 Department of Medical Biometry and Epidemiology, University Medical Center Hamburg-Eppendorf, Martinistraße 52, 20246 Hamburg, Germany

**Keywords:** Activities of daily living, Constraint-induced movement therapy, Occupational therapy, Participation, Physical therapy, Stroke

## Abstract

**Background:**

Stroke leads to constant rehabilitation needs even at the chronic stage. However, although many stroke patients receive physical or occupational therapy in primary health care, treatment prescriptions do not generally specify therapeutic goals; in particular, participation is not established as an explicit therapeutic goal in the ambulatory setting. The primary aim of this study is to evaluate the efficacy of a therapy regimen for chronic stroke patients (modified ‘constraint-induced movement therapy (CIMT) at home’) with impaired hand or arm function with regard to the prerequisites of participation in everyday activities: a sufficient arm and hand function. ‘CIMT at home’ will be compared with conventional physical and occupational therapy (‘therapy as usual’).

**Methods/design:**

The study is a parallel cluster randomized controlled trial with therapy practices as clusters (*n* = 48). After written consent from the patients (*n* = 144), the therapists will be randomly assigned to treat either the intervention or the control group. Blinded external assessors will evaluate the patients using standardized outcome measures before and after the intervention, and six months later. The two coprimary endpoint assessments of arm and hand function as prerequisites for participation (defined as equal involvement in activities of daily living) are the motor activity log (quality of arm and hand use) and the Wolf motor function test (arm and hand function). These assessments are made four weeks post-treatment and relativized to baseline performance. Changes in primary outcomes will be analyzed with mixed models, which consider the hierarchical structure of the data and will be adjusted to the baseline measurements and sex. The primary analysis will be the comparison of the two randomized groups, with respect to the adjusted averages for each of the two coprimary endpoints. To keep an overall significance level of 5%, the two endpoints will be tested at the significance level of 5% each in hierarchical order.

**Discussion:**

A modification of the CIMT, feasible in the patients’ homes (CIMT at home), appears to be a promising therapeutic approach in the ambulatory care of chronic stroke patients. With proven efficacy and practicality, a participation-oriented, stroke-specific treatment would be available in primary care.

**Trial registration:**

ClinicalTrials.gov NCT01343602

## Background

Stroke is the fourth leading cause of death in Germany [1] and the most common cause of lifelong disability in adults [2]. In the chronic phase, that is, six months or more after the event, the aim is to maintain and, if possible, further improve the achieved results of rehabilitation [3]. Apart from a variety of other problems, many stroke patients suffer from impaired arm and hand function, which often affects their participation in everyday activities. In the context of health, participation is defined as involvement in a life situation and is related to the execution of a task or action (activity) [4]. Therapeutic approaches aimed at enhancing participation involve exercises that address the demands of daily life, in an environment that at least resembles the patient’s individual environment at home. Correspondingly, since 2001 in Germany, rehabilitation services are required by law to prioritize participation as a treatment goal, rather than merely providing social care [5]. Despite this, many stroke patients who receive physical or occupational therapy in the primary health care setting are provided with therapy prescriptions that specify neither the appropriate therapeutic approach nor the therapeutic goal. So far, there have not been any therapeutic concepts concentrating solely on enhancing patients’ participation. Recent developments in treatment, such as arm ability training, constraint-induced movement therapy (CIMT), and robot-assisted arm rehabilitation, focus on the improvement of activities of daily living as prerequisites of participation. These concepts are recommended by guidelines [6], since they have shown evidence of good therapeutic results [7] in terms of motor function improvement and improvements to the dexterity of the affected arm, as well as a clinically relevant effect on the usage of the affected arm during activities of daily living. Whereas these therapies are now increasingly being used in in-patient rehabilitation, they have not yet been established in ambulatory care (for example, outpatient physical and occupational therapy) and are not listed in the German catalogue of care interventions and modalities (*Heilmittelkatalog*) [8]. In testing CIMT in an ambulatory setting, this project provides a contribution towards an explicit participation-oriented approach to treating stroke patients in ambulatory care.

### Novel aspects of HOMECIMT

Constraint-induced movement therapy is a therapy shown to be effective in the treatment of stroke patients largely independent of the post-stroke time [9-11]. In addition, lasting therapeutic effects have been demonstrated after the completion of therapy [12]. However, in this regard, the literature is inconclusive. A Cochrane review [13] and its update [14] found no evidence of persisting benefits on the outcome disability, while a more recent review [15], which focused on the effect of CIMT and modified CIMT on activity and participation, describes evidence on hand mobility. Both reviews recommend further randomized trials with larger sample sizes, relevant measures and a sufficient follow-up. Therefore, HOMECIMT aims to address these requests in the conception of the trial.

Constraint-induced movement therapy is suitable for stroke patients with mild to moderate impairment of their arm and hand function. Among the core elements are repetitive, task-oriented and daily-life-oriented exercises, ‘shaping’ (that is, training demands that are increased according to patients’ current performance limits) and the immobilization of the less affected hand or arm [10]. Patients are actively involved in treatment planning and delivery. By formulating participation-oriented goals, patients take personal responsibility for their treatment. Thus, the approach includes elements of participatory care. Constraint-induced movement therapy is recommended by the German Society of Neurology as an effective treatment that is superior to conventional physical therapy methods [6]. Despite this, CIMT has not yet been established as a treatment option in statutory health care.

At the University Hospital Hamburg-Eppendorf, CIMT has been used successfully for the past 10 years [16-18]. In a feasibility study, we showed that the modified concept ‘CIMT at home’, which is delivered at patients’ own homes, is feasible and achieves similar results to the ‘classic’ CIMT concept [19]. An innovative aspect of ‘CIMT at home’ is the active involvement of a non-professional coach (for example, a family member) to support the patient in the therapeutic process. Patients are encouraged to practice their exercises under the guidance of a non-professional coach for two hours a day over the course of four weeks. The therapist draws up the exercise program together with the patient, instructs the patient and coach in how the exercises should be performed, and carries out weekly home visits to supervise the treatment progress.

Thus, the modified CIMT concept trains the increased use of the affected arm in daily life and in the unique home environment, so as to enhance one of the prerequisites for improving participation in everyday activities. So far, no study has compared the use of CIMT in an ambulatory setting with conventional physical and occupational therapy. In view of this research gap, this randomized controlled trial was designed to evaluate the efficacy of a modified approach to constraint-induced movement therapy (‘CIMT at home’) for chronic stroke patients, compared with conventional physical and occupational therapy (‘therapy as usual’) in terms of their enhancement of activities of daily living as one of the prerequisites for participation. The target group of this study consists of chronic stroke patients with an indication for physical or occupational therapy.

## Methods/design

### Objectives

The primary objective of this study is to evaluate the efficacy of a modified approach of constraint-induced movement therapy (‘CIMT at home’) for chronic stroke patients with impaired hand or arm function, compared with conventional physiotherapy and occupational therapy (‘therapy as usual’) with regard to the prerequisites of participation in everyday activities: that is, to attain a sufficient arm and hand function.

Secondary objectives are to explore the effects on the primary endpoint measures six months post-intervention, as well as the effects the treatment has on stroke-related quality of life, activities of daily living, amount of arm and hand use, and function and dexterity of the fingers on the more affected hand or arm, both at the end of the intervention and six months later. Additionally, we will determine health service utilization and costs.

### Study design

**Figure 1:**
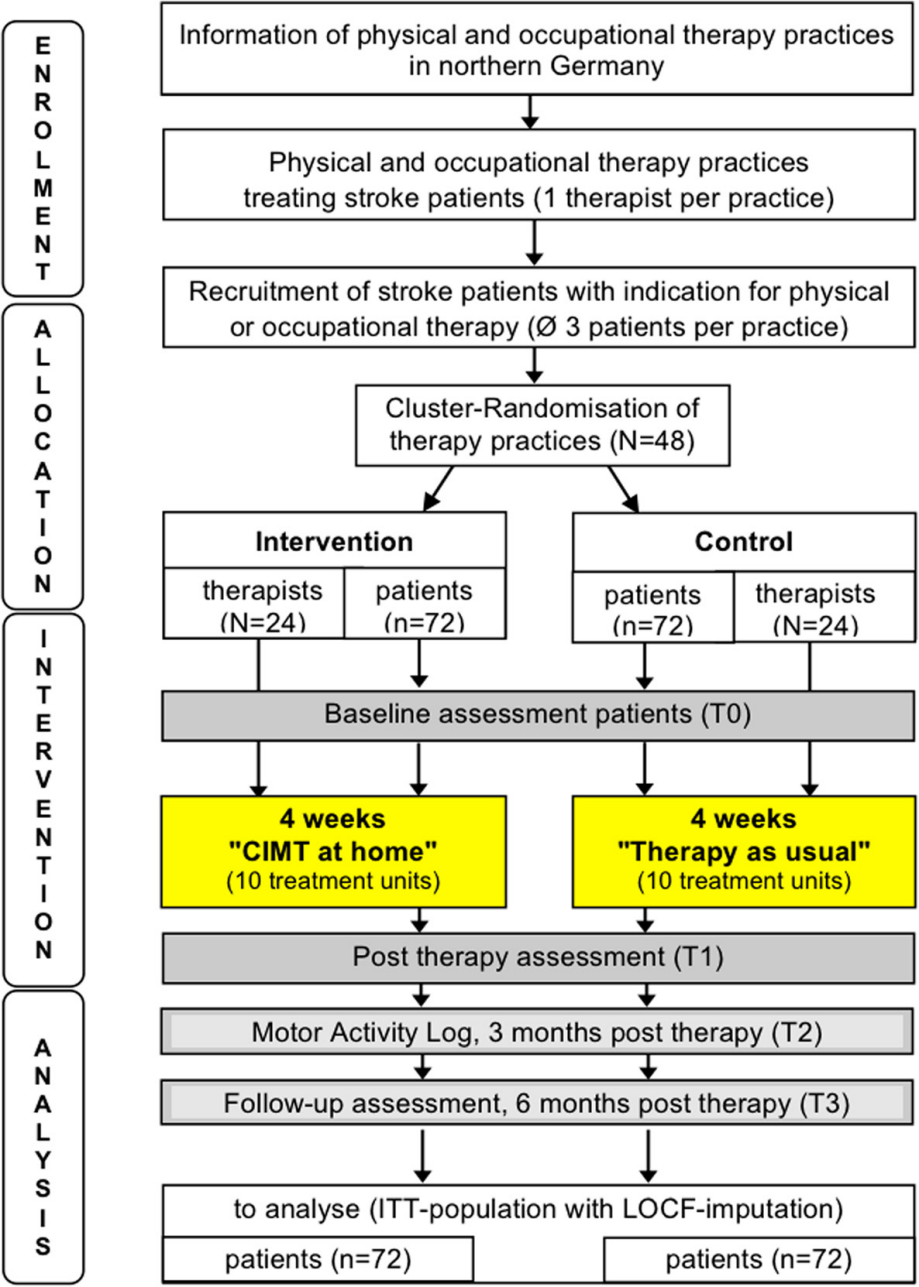
**Design of the HOMECIMT trial.** Flowchart showing enrolment, allocation, follow-up, and analysis for the HOMECIMT trial participants with regard to the primary endpoint. ITT, intention-to-treat; LOCF, last-observation carried forward.

The HOMECIMT study is designed as a two-armed, cluster randomized trial with physical and occupational practices as clusters (see Figure 1). The central randomization is stratified by region and takes place at the practice level, to avoid contamination effects from personal contacts. Owing to the nature of the intervention, neither the therapists nor the patients can feasibly be blinded. However, the assessors will be blinded during the data collection. The patients’ recruitment practices are randomized to either the intervention group (‘CIMT at home’) or the control group (‘therapy as usual’) and treat the enrolled patients according to their allocation. On the patient level, all primary and secondary parameters will be measured at baseline (T0), at the end of the four-week intervention (T1) and six months after completing the treatment (follow-up, T3). In addition, patients will fill in a motor activity log (MAL) to assess quality of movement, a EuroQoL EQ-5D form, and a health service utilization questionnaire three months after the end of intervention (T2).

### Sample size

The sample size was calculated for the two primary outcome measures quality of movement, as measured by the motor activity log (MAL-QOM), and performance time in the Wolf motor function test (WMFT-PT). For the first primary endpoint (MAL-QOM), an effect size of 0.5 was pursued based on the reported minimal clinically important improvement of 0.50 on a scale from 0 to 5 [20]. With a power of 80% and an α-error probability of 5%, 2 × 64 patients need to be included if the randomization occurs at the patient level. With an assumed intra-cluster correlation of 0.05 and 3 included patients per cluster, a design effect of 1.1 was calculated, increasing the sample size to 72 patients and 24 practices per group.

For the second primary endpoint (WMFT-PT) an effect size of 0.55 was calculated based on the minimal clinically important improvement of 0.60 on the logarithmic performance-time scale [21]. With identical α and β design effects, a sample size of 63 patients and 21 practices per group was calculated. Since the first coprimary endpoint requires a larger sample size, the sample size was chosen accordingly. We decided to approach 60 practices for participation in the study, expecting at least 48 to take part, which allows us to demonstrate effect sizes of 0.49 and greater for the second primary endpoint.

### Recruitment of practices and patients

All physical and occupational therapy practices treating stroke patients are eligible to participate in the study. Because the study center is located in Hamburg, recruitment is performed consecutively in selected areas of northern Germany that are sufficiently accessible for the study personnel from Hamburg as well as for local therapy practices. Based on community numbers (geographical recruitment) we formed an area of eight related regions, each within 50 km of a city. All therapy practices with known addresses are invited to an information meeting nearby. Additionally, written information about the study is offered. The study design allows the inclusion of one therapist per practice.

The referrals to the study follow two steps to allow all eligible patients access to the study. In a first step, active practices willing to participate are asked to create a list of all stroke patients currently being treated (incident and prevalent cases, regardless of prior treatment and duration of therapy). To identify all potentially eligible patients, the inclusion and exclusion criteria are discussed with the study team for each listed patient. All patients meeting the inclusion criteria obtain written study information from their therapists and are asked to meet study personnel for more information about the study and to provide informed consent (second step). We aim to include an average of three patients per practice. The study center records the recruitment process for each therapy practice, including the names of the study team, the date, and the result of the patient visit (whether the patient enrolls or not). Document and data completeness are checked by a second member of the study team, who also ensures that all data forms are returned to the study center. To maintain participants in the study, we provide good accessibility (telephone hotline, email, and fax) to the study personnel, who respond to any questions and emerging issues during the study period.

### Randomization

Therapy practices are randomly allocated to either ‘CIMT at home’ or ‘therapy as usual’ in a 1:1 ratio, stratified by region. Following the recruitment of patients, a research assistant (from the Department of Medical Biometry and Epidemiology, University Hospital Hamburg-Eppendorf), who is not involved in the project, performs the randomization consecutively for each region. Therapy practices are informed of their group allocation in writing by the study center. Patients will be entered into either the intervention group or the control group based on the study group allocation of their treating practice.

### Inclusion and exclusion criteria

#### Practice inclusion and exclusion criteria

Practices are eligible if they currently treat three or more patients who meet the inclusion criteria. Participating therapists are required either to hold a professional qualification, relevant to the treatment of stroke patients (for example, the Bobath course), or have at least two years’ experience in the treatment of stroke patients. All participating therapists have to provide written informed consent that they meet, and will adhere to the study requirements and procedures. Practices are excluded if they already offer CIMT as a therapy option and if another therapist in the practice is already participating in the study.

#### Patient inclusion and exclusion criteria

Patients are eligible for inclusion in the study if they have had a stroke leading to upper extremity hemiparesis with mild to moderate impairment of hand or arm function at least six months prior to study enrolment (minimal residual hand function of at least 10° active wrist extension, at least 10° active thumb abduction or extension, and at least 10° extension of two additional fingers). Further inclusion criteria are: an indication and a prescription for physical or occupational therapy, age ≥ 18 years, and the availability of a non-professional coach (for example, a family member).

Patients meeting the following criteria are excluded: insufficient ability to communicate in German, severe impairment of verbal communication ability (for example, severe aphasia), inability to consent (for example, dementia), severe neurocognitive deficits (mini-mental status test <23), terminal illness, life-threatening comorbidities, previous treatment using CIMT, or simultaneous participation in another treatment study targeting stroke recovery.

### Data collection

After obtaining written informed consent, therapists and patients are registered in the study center of the Department of Primary Medical Care, Hamburg (Germany), which is responsible for administration, coordination, data management, and monitoring (including database-set up and validation, data entry, coding, and query management).

Therapists are asked to fill in a questionnaire with information about their personal details and those of their practice. During the four-week intervention, therapists document the treatment of each study patient, including information about any additional therapies (for example, speech therapy, occupational therapy, and physiotherapy) as well as any adverse events, such as illness or hospitalization, as well as any vacations. To assure sufficient data quality, all participating therapists will be monitored by telephone using a standardized documentation sheet.

Patient data collection will be performed by blinded assessors during home visits conducted at baseline (T0), after the intervention (T1), and at the six-month follow-up (T3). In addition, an intermediate telephone interview will be conducted at the three-month follow-up (T2). All assessors receive training before they start collecting data and refresher courses will be offered during the study. The study center provides the assessors with standardized paper-based interview forms and test materials. The assessors are blinded to the patients’ group affiliation. They have no access to study computers and documents, and are not involved in any other study aspects, apart from the data collection in the patients’ homes. To keep assessors blinded, patients and non-professional coaches are prompted to give no information to the assessors, neither about their treatment nor about other experiences from participating in the study.

### Outcome measures

#### Primary outcome

We do not know of any generally accepted direct measure of participation, particularly not for people with impaired hand or arm function. Therefore, we use two subscores, MAL-QOM and WMFT-PT as coprimary outcomes; these measure activity and motor function of the impaired hand and arm as important prerequisites for participation in activities of daily living. Both measurements are customarily used in trials with CIMT and will be measured four weeks post-treatment relative to the baseline performance.

In the MAL-QOM, patients assess quality of movement for 30 daily activities on a scale from 0 (= no use of hand or arm) to 5 (= normal usage), focusing on the change in quality of arm and hand use. In the WMFT-PT, the average time in seconds needed to complete 15 tasks is measured by an assessor to evaluate changes in arm and hand function.

#### Secondary outcomes

The following parameters are secondary outcome measures taken four weeks post-treatment and at the six-month follow-up (relative to the baseline performance): the amount of arm usage, as rated in the MAL), the functional ability of the hand and arm (measured using the Wolf motor function test), finger dexterity (the nine hole peg test) [22], generic quality of life (EuroQoL form, EQ-5D) [23] and stroke-related quality of life (measured on the Stroke Impact Scale) [24], independence in daily life (Barthel Index) [25], and instrumental activities of daily living [26]. In addition, the following patient information is collected during the baseline assessment to allow for adjustment of any treatment effects: sex, age, depression (measured using the PHQ-9) [27], comorbidity [28], pain [29], neuropsychological deficits (measured using the mini-mental status test, MMST) [30], and level of education. Furthermore, health care utilization and total health care costs will be calculated.

To study the impact of cluster characteristics on the main outcomes, the therapists’ ages, sex, and professional experience, as well as data on the sizes of the practices (number of patients and treatment units per year, and number of stroke patients treated in the last 12 months) will be obtained.

Quantitative and qualitative process evaluation will be conducted at the end of the study: the therapists, patients, and non-professional coaches of the intervention groups will be asked about their acceptance of ‘CIMT at home’ through a questionnaire or structured interview. Therapists will report on their experiences of ‘CIMT at home’ in focus groups, with a special focus on supporting and limiting factors.

### Data analysis

Data will be analyzed according to the CONSORT statement extension for cluster randomized trials [31]. A detailed statistical analysis plan will be prepared and finalized before the code is broken. This is a cluster randomized trial with two treatment arms to confirm the two-tailed hypothesis: ‘CIMT at home’ (intervention) is superior to ‘therapy as usual’ (control). The intention-to-treat (ITT) analysis of primary data will be based on the available clinical data from all randomized patients (MAL-QOM) or blinded assessors (WMFT-PT) at T1 (after the end of the four-week intervention), and on the characteristics of the clusters (practices). In case of missing follow-up values, a last-observation carried forward (LOCF) imputation will be performed, that is, the baseline determination will be imputed as follow-up determination. For each of the two primary endpoints, a linear mixed model will be calculated for the difference between the intervention and control groups at T1. Group and region will be considered fixed effects while practice will be considered a random effect under control of the baseline covariates (baseline values of the specific outcome, age, depression (PHQ-9), comorbidity, neuropsychological deficits (result of MMST), educational level), and sex of patient and therapist. The two primary endpoints will be tested hierarchically at the two-sided α-level of 0.05. First, the first primary endpoint (MAL-QOM) will be tested and, if a significant difference is detected, the second primary endpoint (WMFT-PT) will be analyzed at the same test level. Missing values will not be replaced for the primary analysis. However, sensitivity analyses will be performed with different methods of missing value imputation to study the robustness of the findings. An additional analysis will be conducted on a per-protocol analysis set.

Only the result of this primary efficacy analysis will be interpreted in a confirmatory manner.

The secondary endpoints will be examined in an exploratory manner with appropriate procedures, including subgroup analyses of patients and therapists according to their sex. Analyses of secondary endpoints should provide an indication on the consistency of the results from the evaluation of primary endpoints. The process evaluation will be carried using qualitative interviews and standardized questionnaires for participants and therapists.

Interim analyses are not planned. Statistical analyses will be carried out with SAS, Version 9.3 (SAS Institute, Cary, NC, USA).

### Intervention

Patients in the intervention group receive ‘CIMT at home’ , a complex intervention based on a modification of CIMT for the use in the patient’s individual home environments.

In the original technique [32], CIMT is administered by a therapist (physical, occupational) or psychologist for six hours, every weekday, over the course of two weeks. Additionally, patients wear a resting hand splint on their unaffected arm, aiming to prevent use of that arm for about 90% of waking hours. Overall, about 60 hours of professional training and supervision are provided. The CIMT core elements are: active, repetitive training, exercises relevant to everyday life, participative goal attainment, adaptation to the patient’s options (shaping), and restriction of the less affected arm.

Based on our long-standing experience with the original CIMT, we developed the modification ‘CIMT at home’ , which is applicable in the patients’ homes with less professional effort. In a previous feasibility study, we showed that ‘CIMT at home’ was not only feasible but also as effective as the original technique [19]. In contrast with the original technique, ‘CIMT at home’ includes 40 hours of training over the course of four weeks and is performed in the patients’ homes, together with a non-professional coach (for example, a family member). Once a week, the therapy is supervised by a physical or occupational therapist. Thus, about five hours of professional supervision are provided.

### Course of the intervention

**Figure 2:**
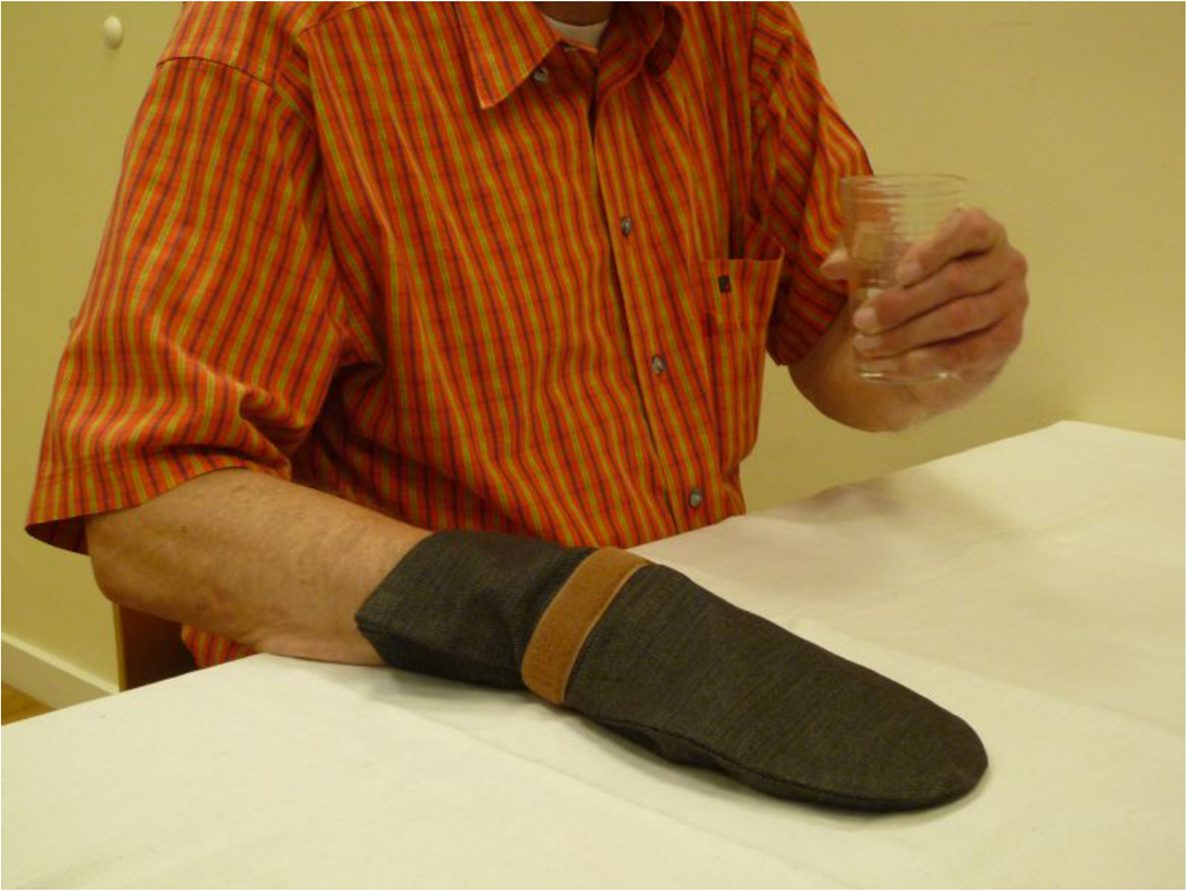
**Therapy concept.** This figure illustrates one of the key therapeutic principles of CIMT: restriction of the less affected hand induces the patient to use the affected arm to drink a glass of water as a typical activity of daily living.

During an initial home visit, therapists will determine (together with the patients) an individually tailored home training program, focusing on everyday practice and will instruct non-professional coaches. Over the course of four weeks, that is, 20 consecutive days (no training is required on weekends), the patients will train for two hours a day, together with their instructed non-professional coach (for example, a family member) at home. During this time, the patients and their coaches will be applying shaping techniques. Patients are instructed to wear a resting mitten (see Figure 2) for four to six hours a day during the entire treatment period, and are encouraged to keep a training diary to document their daily exercises, times of training and periods of mitten wearing. A review of the training diaries will allow quality control by verifying whether the therapy was carried out in accordance with the ‘CIMT at home’ concept. During the four-week intervention, therapists will provide weekly home visits to instruct the patients and to supervise the training (five home visits in total, equal to ten treatment units or 250 to 300 minutes). To support the therapists in delivering this new therapeutic concept, an expert telephone hotline will be provided.

### Training of therapists

Physical and occupational therapists receive training in the modified therapy approach ‘CIMT at home’, including: (1) information on the theoretical background of CIMT, (2) the principles of CIMT, (3) the therapeutic concept of ‘CIMT at home’ (cognitive preparing, restriction of motion, motor training, shaping techniques (practice routine, motivation), the schedule of the four-week intervention (home visits 1 to 5), documentation (target and therapy agreements, training, activities of daily living, and wearing the mitten)), and (4) training in the selection of suitable exercises relevant to everyday life.

### Control

Patients in the control group receive the usual care (‘therapy as usual’) dose-matched to the intervention group treatments (10 treatment units or 250 to 300 minutes). ‘Therapy as usual’ consists of a variety of therapy options for stroke patients (for example, Bobath, Vojta, or proprioceptive neuromuscular facilitation) usually provided by occupational or physical therapists. Therapy sessions take place in the patients’ homes or at the therapists’ practices. Therapists in the control group apply ‘therapy as usual’ in accordance with the present prescription and their individual therapy knowledge and preferences. Hence, it is possible that patients of the control group receive different types of therapy (for example, Bobath, Vojta, or proprioceptive neuromuscular facilitation) during their four-week treatment course. This is a common scenario in therapeutic practice and will, therefore, be tolerated in the study. In any case, all patients will receive the same amount of treatment (that is, 10 treatment units or 250 to 300 minutes).

The therapists will receive written instructions on the course of therapy for their study patients. During the four-week intervention phase the therapists will document the performed therapy sessions, as well as any agreements (for example, homework) made with patients using a standardized documentation sheet. To motivate the control group therapists to participate in the study, they will be offered the ‘CIMT at home’ training after the end of the follow-up phase.

### Monitoring of potential unintended harms and adverse events

During study participation, hospitalizations and severe diseases will be documented as well as any adverse events. Regarding the therapy, no specific adverse events are expected, since the indication for physiotherapy or occupational therapy is mandatory for all study participants. Nevertheless, potential unintended harms may include falls, frailty, and severe depression. In addition, patients of the intervention group may perceive some of the CIMT specific conditions (for example, the daily exercise program or the wearing of a special mitten) as stressful. Thus, all study participants (patients, non-professional coaches, therapists, and assessors) will be encouraged to report any events. Feedback and details of events will be documented and evaluated at the end of the study.

### Quality control and quality assurance

The data management center is at the University Medical Center Hamburg-Eppendorf. A trial steering committee composed of the principal investigator, experts in the field of CIMT and the data management center is responsible for all decisions concerning the conduct of the study. The trial’s study team (Department of Primary Medical Care, University Medical Center Hamburg-Eppendorf) will be responsible for monitoring the trial. Study employees will regularly contact the trial sites (therapists and assessors), to ensure that the rights of the trial participants are protected, the study data is documented correctly and completely, and that the trial is conducted in accordance with the study protocol and complies with good clinical practice and legal requirements at the trial site. A scientific advisory board supports the study management in scientific questions concerning study design, implementation, and evaluation.

### Ethics and legal aspects

The study is being conducted in accordance with the Helsinki Declaration and the ICH Guidelines for Good Clinical Practice. The study team is committed to performing all study procedures in accordance with the standards of good clinical practice. All participating therapists and assessors will be informed of their responsibilities by the study team. The study protocol was approved by the Ethics Committee of the Medical Association of Hamburg in April of 2011 and was amended in July of 2011 (Approval-No. PV 3737). It has been registered under http://www.clinicaltrials.gov (NCT01343602).

### Study timeframe

**Figure 3:**
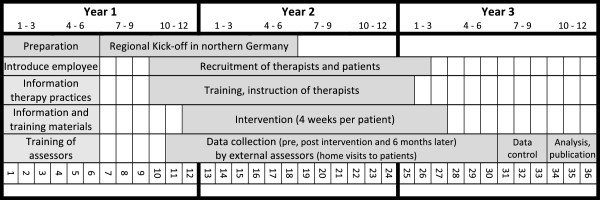
Timeline of the HOMECIMT trial.

The study’s duration totals three years, (see Figure 3). The recruitment of the therapy practices started in July 2011 (first region) and was finished in January 2013. The recruitment of patients started in September 2011 (first patient in) and ended in March 2013 with the last patient-out date set for 1 October 2013. The central randomization is stratified by region as soon as all patients of the region are included. The training of assessors is also organized regionally. Data collection is conducted consecutively according to the regional recruitment strategy.

## Discussion

In the ambulatory care of chronic stroke patients, a therapy approach with a specific focus on participation is lacking. Constraint-induced movement therapy provides an evidence-based, effective, and activity-oriented treatment for stroke patients. Our previous feasibility study showed that the modified CIMT program ‘CIMT at home’ is an effective treatment option for chronic stroke patients with motoric hand or arm problems in ambulatory care [19]. The HOMECIMT project is designed to show whether ‘CIMT at home’ is superior to ‘therapy as usual’ with regard to its enhancement of patients’ activities of daily living as prerequisites of participation in everyday activities. If ‘CIMT at home’ proves the superior treatment, an explicit participation-oriented therapy for stroke patients in ambulatory care would be available. In this case, ‘CIMT at home’ could be readily implemented in routine care, since its practical implementation has already been considered in the development of the program and the concept takes existing resources into account. If necessary, results from the focus group sessions with the intervention group therapists will be used to improve and further develop the concept. In line with the CONSORT statement, the results of this trial will be presented in the context of a systematic review of the existing randomized controlled trial evidence.

### Trial status

Recruitment was completed in March 2013.

## Abbreviations

CIMT: Constraint-induced movement therapy; ITT: Intention-to-treat; LOCF: Last-observation carried forward; MAL: Motor activity log; MAL-QOM: Quality of movement, as measured by the motor activity log; WMFT-PT: Performance time in the Wolf motor function test.

## Competing interests

The authors declared that they have no competing interests.

## Authors’ contributions

AB, GK, and KW conceived the study. HK, KH and BT participated in implementing the study. AB wrote the first draft of the manuscript, MS, GK, AD, and KW revised it. All authors commented on the draft and approved the final manuscript.
